# Supplementary material for: Early conservation benefits of a de facto marine protected area at San Clemente Island, California
Source: PLoS One. 2020 Jan 16;15(1):e0224060. doi: 10.1371/journal.pone.0224060 (PMC6964903; doi:10.1371/journal.pone.0224060)
Supplement: S1 Table — (DOCX) [file pone.0224060.s001.docx]

| Focal species | a | b | Reference | Notes |
| --- | --- | --- | --- | --- |
| Lingcod (*Ophiodon elongatus*) | 0.01330 | 3.0000 | Fishbase |  |
| California sheephead (*Semicossyphus pulcher*) | 0.02890 | 3.0000 | Fishbase |  |
| California scorpionfish (*Scorpaena guttata*) | 0.03300 | 2.9960 | Fishbase |  |
| Ocean whitefish (*Caulolatilus princeps*) | 0.02390 | 3.0000 | Fishbase |  |
| Bocaccio rockfish (*Sebastes paucispinis*) | 0.01321 | 3.0000 | Fishbase |  |
| Copper rockfish (*Sebastes caurinus*) | 0.01746 | 3.0000 | Fishbase |  |
| Olive/yellowtail rockfish (*Sebastes serranoides/S. flavidus*) | 0.01080 | 2.9680 | Fishbase |  |
| Vermilion/Canary rockfish (*Sebastes miniatus/S. pinniger*) | 0.03270 | 3.0000 | Fishbase | parameters for *S. miniatus* |
| Dwarf-red rockfish (*Sebastes rufianus*) | 0.01464 | 2.9840 | Love et al. 1990 | borrowed from *S. hopkinsi* |
| Halfbanded rockfish (*Sebastes semicinctus*) | 0.01900 | 2.8100 | Fishbase |  |
| Squarespot rockfish (*Sebastes hopkinsi*) | 0.01464 | 2.9840 | Love et al. 1990 |  |
| Sanddab *(Citharichthys* spp.) | 0.00776 | 3.0757 | Gartz 2004 | parameters for *C. stigmaeus* |
| Surfperch (Embiotocidae, multiple species) | 0.06160 | 2.8640 | FishBase | parameters for *E. jacksoni* |

**S1 Table. Length-weight relationship parameter values and sources for focal fish species.**
