# Supplementary material for: Early conservation benefits of a de facto marine protected area at San Clemente Island, California
Source: PLoS One. 2020 Jan 16;15(1):e0224060. doi: 10.1371/journal.pone.0224060 (PMC6964903; doi:10.1371/journal.pone.0224060)
Supplement: S2 Table — (DOCX) [file pone.0224060.s002.docx]

**S2 Table. Variables (name, year, site, percent rock, mean depth, and area surveyed) associated with ROV transects.**

| Name | Year | Site | Percent rock | Mean depth (m) | Area surveyed (m^2^) |
| --- | --- | --- | --- | --- | --- |
| SCF01 | 2012 | fished | 32.23 | 48.30 | 926.00 |
| SCF02 | 2012 | fished | 54.20 | 43.80 | 912.00 |
| SCF03 | 2012 | fished | 68.83 | 99.80 | 895.00 |
| SCF04 | 2012 | fished | 61.16 | 83.00 | 917.00 |
| SCF05 | 2012 | fished | 61.31 | 66.60 | 1045.00 |
| SCF06 | 2012 | fished | 74.22 | 47.90 | 792.00 |
| SCF50 | 2013 | fished | 74.45 | 57.00 | 857.00 |
| SCF51 | 2013 | fished | 55.62 | 78.60 | 1276.45 |
| SCF52 | 2013 | fished | 75.30 | 97.50 | 1279.92 |
| SCF55 | 2013 | fished | 0.00 | 102.10 | 1244.87 |
| SCF60a | 2013 | fished | 82.64 | 83.20 | 371.12 |
| SCF60b | 2013 | fished | 82.60 | 78.20 | 411.31 |
| SCF60c | 2013 | fished | 49.83 | 74.20 | 482.25 |
| SCF53 | 2013 | fished | 0.00 | 114.40 | 1205.81 |
| SCF54 | 2013 | fished | 0.00 | 108.70 | 977.65 |
| SCG01 | 2012 | DFMPA | 65.14 | 56.90 | 1697.00 |
| SCG02 | 2012 | DFMPA | 85.71 | 41.80 | 1019.00 |
| SCG03 | 2012 | DFMPA | 78.98 | 42.50 | 763.00 |
| SCG04 | 2012 | DFMPA | 61.04 | 50.70 | 2393.00 |
| SCG05 | 2012 | DFMPA | 42.94 | 73.80 | 1926.00 |
| SCG06 | 2012 | DFMPA | 52.84 | 53.10 | 762.00 |
| SCG07 | 2012 | DFMPA | 29.81 | 53.10 | 775.00 |
| SCG50 | 2013 | DFMPA | 26.30 | 87.00 | 1350.03 |
| SCG51 | 2013 | DFMPA | 59.21 | 71.20 | 2274.02 |
| SCG52a | 2013 | DFMPA | 80.73 | 47.90 | 924.70 |
| SCG52b | 2013 | DFMPA | 58.98 | 67.90 | 1638.55 |
| SCG60 | 2013 | DFMPA | 0.00 | 101.40 | 1674.68 |
| SCG62 | 2013 | DFMPA | 0.00 | 111.80 | 774.50 |
| SCG57 | 2013 | DFMPA | 75.49 | 91.90 | 1942.82 |
| SCG58 | 2013 | DFMPA | 54.21 | 85.90 | 696.73 |
| SCG55 | 2013 | DFMPA | 56.20 | 153.00 | 1423.70 |
| SCG56 | 2013 | DFMPA | 71.38 | 201.40 | 1189.34 |
| SCG53a | 2013 | DFMPA | 75.22 | 63.50 | 1288.85 |
| SCG53b | 2013 | DFMPA | 65.35 | 46.80 | 751.09 |
