# Supplementary material for: Early conservation benefits of a de facto marine protected area at San Clemente Island, California
Source: PLoS One. 2020 Jan 16;15(1):e0224060. doi: 10.1371/journal.pone.0224060 (PMC6964903; doi:10.1371/journal.pone.0224060)
Supplement: S3 Table — (DOCX) [file pone.0224060.s003.docx]

**S3 Table. Means and standard errors for focal species density at fished and DFMPA sites.**

|  | Fished |  | DFMPA |  |
| --- | --- | --- | --- | --- |
|  | Density (organisms/m^2^) | SE | Density (organisms/m^2^) | SE |
| Rock-associated focal species |  |  |  |  |
| Predatory fishes |  |  |  |  |
| Lingcod | 6.07E-04 | 2.43E-04 | 5.35E-04 | 2.56E-04 |
| California sheephead | 5.37E-03 | 2.43E-04 | 2.19E-02 | 7.23E-03 |
| California scorpionfish | 5.11E-04 | 3.17E-04 | 1.37E-04 | 9.41E-05 |
| Ocean whitefish | 1.23E-03 | 1.09E-03 | 1.65E-03 | 7.02E-04 |
| Bocaccio rockfish | 4.26E-03 | 1.67E-03 | 4.74E-03 | 1.10E-03 |
| Copper rockfish | 1.67E-03 | 6.87E-04 | 1.11E-03 | 1.10E-03 |
| Olive/yellowtail rockfish | 1.49E-03 | 1.25E-03 | 3.14E-03 | 1.10E-03 |
| Vermilion/canary rockfish | 2.06E-03 | 7.04E-04 | 1.93E-03 | 1.10E-03 |
| Dwarf rockfishes |  |  |  |  |
| Dwarf-red rockfish | 2.11E-02 | 1.24E-02 | 1.12E-02 | 7.52E-03 |
| Halfbanded rockfish | 6.99E-02 | 2.47E-02 | 3.87E-02 | 1.45E-02 |
| Squarespot rockfish | 2.30E-01 | 1.09E-01 | 3.78E-01 | 1.24E-01 |
| Mobile invertebrates |  |  |  |  |
| California spiny lobster | 7.02E-04 | 4.79E-04 | 2.53E-04 | 2.04E-04 |
|  |  |  |  |  |
| Sand-associated focal species |  |  |  |  |
| Predatory fishes |  |  |  |  |
| Sanddab | 6.07E-04 | 3.30E-04 | 4.01E-04 | 2.75E-04 |
| Surfperch | 3.68E-03 | 1.04E-03 | 3.31E-03 | 9.06E-04 |
| Mobile invertebrates |  |  |  |  |
| California sea cucumber | 3.16E-04 | 2.56E-04 | 3.96E-04 | 1.66E-04 |
