# Supplementary material for: Early conservation benefits of a de facto marine protected area at San Clemente Island, California
Source: PLoS One. 2020 Jan 16;15(1):e0224060. doi: 10.1371/journal.pone.0224060 (PMC6964903; doi:10.1371/journal.pone.0224060)
Supplement: S4 Table — (DOCX) [file pone.0224060.s004.docx]

**S4 Table. Means and standard errors for focal species biomass at fished and DFMPA sites.**

|  | Fished |  | DFMPA |  |
| --- | --- | --- | --- | --- |
|  | Biomass (kg/m^2^) | SE | Biomass (kg/m^2^) | SE |
| Rock-associated focal species |  |  |  |  |
| Predatory fishes |  |  |  |  |
| Lingcod | 4.66E-04 | 1.95E-04 | 5.26E-04 | 1.95E-04 |
| California sheephead | 1.34E-03 | 6.91E-04 | 1.14E-02 | 3.29E-03 |
| California scorpionfish | 2.56E-04 | 1.76E-04 | 1.02E-04 | 7.98E-05 |
| Ocean whitefish | 1.21E-04 | 8.33E-05 | 1.15E-03 | 4.99E-04 |
| Bocaccio rockfish | 3.62E-03 | 2.12E-03 | 3.10E-03 | 8.15E-04 |
| Copper rockfish | 8.14E-04 | 4.51E-04 | 5.72E-04 | 2.50E-04 |
| Olive/yellowtail rockfish | 4.51E-04 | 2.89E-04 | 1.81E-03 | 8.37E-04 |
| Vermilion/canary rockfish | 2.37E-03 | 1.33E-03 | 2.79E-03 | 8.08E-04 |
| Dwarf rockfishes |  |  |  |  |
| Dwarf-red rockfish | 1.68E-04 | 7.74E-05 | 8.64E-05 | 5.48E-05 |
| Halfbanded rockfish | 1.06E-03 | 4.04E-04 | 7.54E-04 | 3.05E-04 |
| Squarespot rockfish | 6.06E-03 | 2.96E-03 | 1.10E-02 | 4.11E-03 |
|  |  |  |  |  |
| Sand-associated focal species |  |  |  |  |
| Predatory fishes |  |  |  |  |
| Sanddab | 1.69E-05 | 9.03E-06 | 3.92E-05 | 3.29E-05 |
| Surfperch | 2.80E-04 | 9.68E-05 | 3.08E-04 | 9.87E-05 |
