# Supplementary material for: Early conservation benefits of a de facto marine protected area at San Clemente Island, California
Source: PLoS One. 2020 Jan 16;15(1):e0224060. doi: 10.1371/journal.pone.0224060 (PMC6964903; doi:10.1371/journal.pone.0224060)
Supplement: Attachment — Submitted filename: PLoS editorial comment_De facto MPAs.docx [file pone.0224060.s005.docx]

**PLoS editorial comment_De factor MPAs**

Overall, a really interesting and well written manuscript. The authors introduce a lot of abbreviations, many of which are used again only once. The PLoS formatting rule is that they should be used three times, otherwise just spell them out.

Line 73: reference not cited corrected. Should be ‘Roberts et al. [ref #]’

Line 76: Don’t capitalise ‘refuge’

Line 77: reference not cited corrected. Should be ‘Rogers-Bennett et al. [ref #]’

Line 96: ‘scuba’ is no longer considered an acronym, but just a regular word, so do not write is all capitals

Line 117: The comma should be a semicolon

Line 118: The comma should be a semicolon

Line 119: The comma should be a semicolon

Line 127: Write as ‘at two sites in the northwest corner’

Line 129: Should spell out ‘SEAL’ here

Line 161: The comma should be a semicolon

Line 162: The comma should be a semicolon

Line 169: The dash should be a comma

Line 179: The abbreviation LWR is not used in the manuscript again so please delete

Line 182: Supporting information is written as ‘S1 Table’ so please correct throughout manuscript and in the supporting information itself.

Line 202: S2 Table

Line 204: The abbreviation FAMD is only used once more so do not introduce it here and just spell it out in the rest of the manuscript.

Also, do not capitalise ‘factor analysis of mixed data’

Line 206: Do not capitalise ‘principal component analysis’

Line 207: Do not capitalise ‘multiple correspondence analysis’

Line 220: The abbreviation GLM is used in several tables but it not defined in the text so please introduce the abbreviation here

Line 225: The semicolon should be a comma

Line 254: S3-4 Tables

Line 276: While AIC has been defined in the text, AICc and AICw have not, so there should be a note for the table explaining what they mean (and for all the tables that use them).

Line 279: Same as above

Line 282: Same as above

Line 296: Same as above

Line 300: delete the duplicate ‘hint at’

Line 310: Replace the dash with a comma and say ‘a ten-fold’

Line 313: I would say ‘fishers’ rather than ‘fishermen’

Line 313: replace the semicolon with a full stop and start the next sentence ‘Since large males….’

Lines 325-326: This is a bit strange and out of place because this is not mentioned in the methods or results and I am not sure why. The authors should mention this comparison was made in the methods and report the result in the results section, rather than here.

Line 368: Do not introduce the abbreviation ‘CBD’ as it is only used once more

Line 369: What is the reference for this quote?

Line 375: Do not introduce the abbreviation ‘OECMs’ as it is only used once more

Line 376: Spell out CBD

Line 377: Spell out OECM
